# Selective Serotonin Reuptake Inhibitors (SSRIs): Effects on male fertility

**DOI:** 10.5935/1518-0557.20240109

**Published:** 2025

**Authors:** Raisa Arruda de Oliveira, Suellen Casado dos Santos, Vera Lúcia de Menezes Lima, Luana Nayara Gallego Adami

**Affiliations:** 1 Embriológica, Pós-graduação, São Paulo, Brazil; 2 Universidade Federal de Pernambuco - UFPE, Recife, Brazil

**Keywords:** Spermatogenesis inhibitors, antidepressant agents, male infertility, semen analysis, sexual dysfunction

## Abstract

Selective Serotonin Reuptake Inhibitors are the most prescribed class of medications in cases of depression. This article discusses the main findings on their effects on male fertility, considering semen parameters and sperm function. This systematic review of the literature delves into the adverse effects of the main SSRIs on male fertility. The software package PRISMA was used to organize the search using keywords related to the research question. The search yielded a total of 125 studies. After the abstracts were read, 18 articles were selected for further analysis. The review ultimately included ten articles about the adverse effects of this class of antidepressants on male fertility, which included decreases in serum testosterone levels, reduced sperm production, decreased sperm reserves in the epididymis, and increased sperm transit time in the tail of the epididymis. The articles included in this review indicated that this class of antidepressants may have adverse effects on male fertility. However, a randomized clinical trial is needed to evaluate the mechanisms of spermatogenic failure and the underlying causes of these effects.

## INTRODUCTION

The recent global rise in mental health issues has led to an increase in the prescription of treatments based on psychotropic drugs. The prevalence of depression grew by 18.4% between 2005 and 2015 to become the third cause of disability (WHO, 2017). Epidemiology studies indicate that depression and anxiety commonly occur in men aged 20 to 60 years (WHO, 2017), a range that encompasses male reproductive age. Given the long-term nature of treatment with antidepressants, men taking this class of medications may suffer from impaired fertility (Boscolo & Crisci, 2022).

Lifestyle changes have caused men and women to work for longer hours, sleep less, suffer from stress, and postpone parenthood (Nascimento & Térzis, 2010). Assisted human reproduction (AHR) and fertility preservation are often considered to address the fertility difficulties couples may eventually face (Nascimento & Térzis, 2010).

Agarwal *et al*. (2021) estimated that infertility affects 8-12% of couples worldwide, with male factor infertility being the primary or contributing factor in approximately 50% of cases. Causes of male subfertility and impaired spermatogenesis vary widely and include congenital (anorchia, cryptorchidism, chromosomal or genetic abnormalities), acquired (varicocele, testicular trauma, germ cell tumors), and idiopathic factors (smoking, drinking, drug use, obesity). Antidepressants may be directly related to male reproductive disorders that alter reproductive parameters (Erdemir *et al*., 2014).

Selective serotonin reuptake inhibitors (SSRIs) are a class of medications commonly prescribed for the treatment of depression, anxiety, and obsessive-compulsive disorders (Edinoff *et al*., 2021). They act on the Central Nervous System at the presynaptic terminals to trigger biochemical reactions in receptors, sensitizing them and increasing serotonin synthesis in the long term (Goodnick & Goldstein, 1998). The half-life of drugs in this class varies between 15 to 24 hours, with fluoxetine being an exception, with a half-life of 96 hours and a therapeutic effect between 2 to 4 weeks (Nelson *et al*., 2007). Antidepressant medications, such as tricyclic antidepressants and SSRIs, can cause all sorts of sexual side effects, including problems with libido, orgasm, and ejaculation (Atmaca, 2020). These medications can elevate serum prolactin levels, thereby suppressing GnRH secretion and decreasing sexual function and semen quality. High levels of prolactin also inhibit the binding of LH to Leydig cells in the testicles, leading to reversible suppression of spermatogenesis. Current data show SSRIs adversely affect semen quality and DNA fragmentation rates and increase oxidative stress in reproductive organs (Beeder & Samplaski, 2020; Xu *et al*., 2022).

SSRIs are the most used class of medications in depression treatment. The present study aimed to discuss the main drugs of this class and the main adverse effects on male fertility, considering semen parameters and sperm function.

## MATERIAL AND METHODS

This systematic review of the literature delves into the adverse effects of the main SSRIs on male fertility. The search for articles included the following descriptors: (male infertility) OR (male fertility) AND (serotonin uptake inhibitor) OR (antidepressant agent) OR (sertraline) OR (fluoxetine) OR (paroxetine) OR (citalopram) OR (escitalopram) OR (fluvoxamine) OR (vilazodone) AND (semen analysis) OR (DNA damage) OR (spermatogenesis) OR (sexual dysfunction), with additional filters to select articles published in the last ten years, randomized controlled trials, clinical studies, clinical trials, case reports, prospective studies, retrospective studies, animal experiments, and systematic reviews. Searches were conducted on EMBASE and LILACS/BVS.

A flowchart was designed in PRISMA 2020 (Page *et al*., 2021) to organize the search process. PRISMA helps design systematic reviews and implement consistent searches, selection, analysis, and synthesis of findings from primary studies to answer a structured research question, decreasing potential methodological biases (Page *et al*., 2021). Inclusion criteria were added to optimize the selection of studies, which included articles written in Portuguese and English, studies about the main SSRIs, studies performed with animal models, and clinical studies. Articles describing alternative models, literature reviews, editorials, and papers about other drugs were excluded. Once the articles related to the study’s central question were selected, the PICO principles (Santos *et al*., 2007) were used to orientate the systematic review and focus on the research question.

According to Santos *et al*. (2007), PICO stands for Population, Intervention (Therapy, Prognosis), Control (or Comparator, in case of absence of intervention, placebo), and Outcome (the expected outcome, what is measured in a population). In this article, P = men of reproductive age on the antidepressants of interest; I = selective serotonin reuptake inhibitors; C = placebo, comparator with the drugs chosen for the research, or psychotropics; O = adverse reactions in male fertility. Within the control group, there was also the inclusion of psychotropics because there were articles with analyses within the parameters relevant to the present research.

## RESULTS

**Figure 1:**
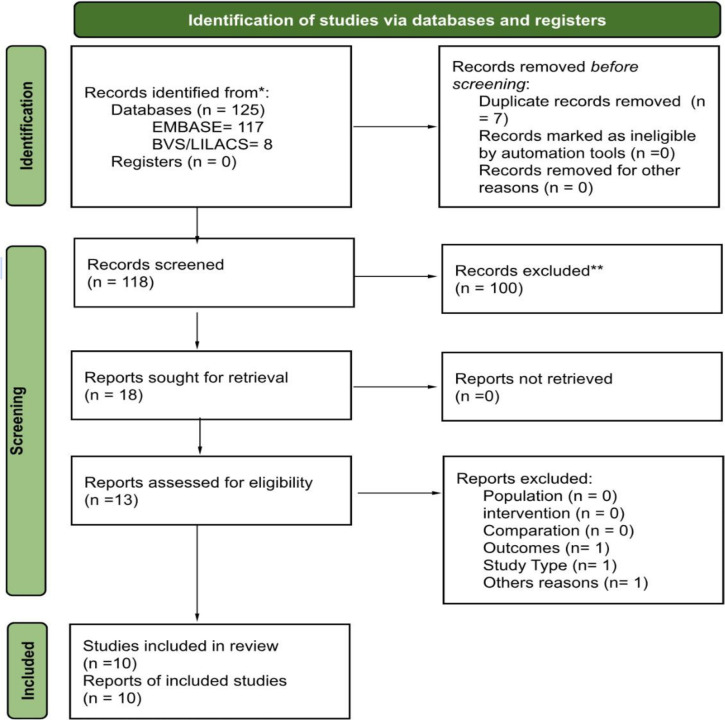
Diagram showing the research flow prepared in the Prisma program (2020).

The search yielded 125 studies (8 LILACS/BVS and 117 EMBASE); seven articles were duplicates (Figure 1). A hundred articles were excluded based on the specified criteria. After the abstracts were read, 18 articles were selected for further analysis. Five of the 18 were excluded because they did not contain significant new data for the present study. Of the remaining 13 publications selected for full-text examination, three were excluded: one due to language (Arabic), another for not explicitly addressing semen parameters and sexual dysfunction, and the last because we could not retrieve its full text. Table 1 summarizes the main findings of interest. The drug vilazodone was not mentioned in the compiled articles.

**Table 1:** Main findings gathered in the present review.

Authors	Participants	Treatment	Main Findings
Akasheh *et al*. (2014)	Men Group 1 Sertraline n=30 (25mg/day) (50mg/day) BT Group n=30 behavioral technique	**EPPb** 1 daily dose of antidepressant or control for 3 months (1 week before, shorter dosage)	**Semen parameters:** significant reduction in sperm concentration (105/mL) and percentage of sperm with normal morphology (*p*<0.05) in Group 1. There was no significant change in semen parameters in patients treated with BT. **FDE^a^** was significantly higher in Group 1 than in the BT Group (*p*=0.004).
Bezerra *et al*. (2019)	Rats *in vitro* Group 1 Sertraline: (1, 3 and 10 µM) Group 2 Fluoxetine: (1, 3 and 10 µM) *in vivo* Group 1 Sertraline (20mg/kg) Group 2 Fluoxetine (20mg/kg)	*in vitro* KCL, phenylephrine or carbachol isolated distal tail of epididymis *in vivo* 1 daily dose of the respective antidepressant or control for 21 days	**Pharmacological parameters:** *in vitro* Groups 1 and 2 harmed the contractions induced by KCl, phenylephrine or carbachol, (> 3 **µM**). Group 2 (1 **µM**) potentiated the contractions induced by phenylephrine. *in vivo* Groups 1 and 2 had an increase in spontaneous contractions of the rat’s distal tail; potentiation of induced contractions were seen with phenylephrine; **Testosterone:** decrease in serum levels in both groups. **Semen parameters:** Groups 1 and 2 had decreased daily sperm production, sperm reserves in the **EP^e^** and **TTE^f^** in the tail of the **EP^e^**.
Câmara *et al*. (2019).	Rats Group 1 Fluoxetine (20mg/kg) Group 2 **GC^c^**	1 daily dose of antidepressant or control for 11 days	**Histopathological analysis:** In Group 1, the frequency of abnormal **TS^d^** increased. The areas of **TS^d^** and **EP^e^**, the number of **TSDA^g^, DNCL^h^**, 17β-HSD6 activity and serum testosterone levels were significantly reduced.
Erdemir *et al*. (2014)	Wistar rats Group 1 Control n=8 Group 2 Sertraline n=8 (10mg/kg) Group 3 Fluoxetine n=8 (10mg/kg) Group 4 Escitalopram n=8 (10mg/kg) Group 5 Paroxetine n=8 (20mg/kg)	1 daily dose of antidepressant or control for 2 months	**LHi:** There were no significant differences between the Groups (*p*=0.092). **FSHj:** Level increased only in Group 2. **Testosterone:** With the exception of Group 3, levels were lower in all groups compared to Group 1. Group 2 had the lowest level compared to Group 1 [40.87 (22.3746.8) *vs*. 15.87 (13.5319.88), *p*<0.01]. **MDA^k^:** There was no significant differences between the Groups (*p*=0.090). **Histological analysis:** The **EJ^l^** was significantly lower in Group 5 compared to Group 1 (*p*<0.05). The negative impact of SSRIs was markedly greater in Group 5.
Galal *et al*. (2016)	Rats Group 1 Fluvoxamine n=12 (9mg/kg-1) low therapeutic dose Group 2 Fluvoxamine n=12 (27mg/kg-1) high therapeutic dose Group 3 **AD^m^** n=12 (0.5ml) Group 4 Fluvoxamine n=12 high and low therapeutic dose Group 5 Fluvoxamine n=12 high and low therapeutic dose	1 daily dose of antidepressant or group 3 for 8 weeks groups 4 and 5 then left without treatment for another 8 weeks GR^n^	**Biochemicals parameters:** Groups 4 and 5 had liver, kidney and heart dysfunction. **Semen parameters:** risk of male infertility, which is indicated by impacts on steroidogenesis hormones and harmful values in the spermogram. **Histological analysis:** high and low therapeutic doses also induced **EO^o^** and **ATT^p^**. The changes observed were reversed, mainly in **GR^n^**
Ilgin *et al*. (2017)	Rats Group 1 Citalopram (5mg/kg) Group 2 Citalopram (10mg/kg) Group 3 Citalopram (20mg/kg)	1 daily dose of antidepressant for 28 days.	**Semen parameters:** Sperm concentration and normal morphology were reduced; **FDE^a^:** increased in Groups administered **CTL^q^** **Histopathological analysis:** changes in the testicles of the rats given **CTL^q^. LH^i^:** increased in groups administered **CTL^q^**. **Testosterone:** increased in all Groups.
Mazzilli *et al*. (2021)	Men Group 1 Antidepressants n=6 SSRI Group 2 Benzodiazepines n=4 Group 3 Antipsychotics n=10 Group 4 Control n=10	1 daily dose of antidepressant or control for 3 months	**Semen parameters:** Group 1 and 3 had semen levels significantly lower than the therapeutic serum range. Sperm concentration and progressive motility were significantly reduced in Groups treated with psychotropic drugs compared to Group 4 (*p*=0.03). These parameters were much lower in Group 3 (*p*<0.05).
Monteiro Filho *et al*. (2014)	Female and baby rats Wistar Group 1 Fluoxetine (5 mg/kg) Group 2 Fluoxetine (10 mg/kg) Group 3 Fluoxetine (20 mg/kg)	1 daily dose of antidepressant given from the 13^th^ day of pregnancy to the 21^st^ day of lactation	**Histopathological analysis:** Group 3 with reduced testicular weight (16%), **EP^e^** (28%), **GS^r^** (18%), **TS^d^** (17%), **VTCL^t^** (30%), **TS^d^** length (17%), daily sperm production (18%). increased **AICL^u^** (7%) and plasma testosterone (49%). Other dosages led to nonsignificant changes.
Moradi *et al*. (2023)	Mice Group 1 Control Group 2 Citalopram (10mg/kg) Group 3 Melatonin (10mg/kg) Group 4 Melatonin (20mg/kg) Group 5 Melatonin (10mg/kg) and Citalopram (10mg/kg) Group 6 Melatonin (20mg/kg) and Citalopram (10mg/kg)	1 daily dose of antidepressant with or without melatonin, for 35 Days	**Semen parameters:** groups treated with melatonin had restored spermatogenesis, improved sperm count, motility, viability, morphology and chromatin integrity. **Testosterone:** improved substantially in Groups 5 and 6. **Histopathological analysis:** improved substantially in Groups 5 and 6. **EO^o^:** increased, however, melatonin restored the antioxidant status, **TAC^v^** levels. Decreased levels of **NO^w^** and **MDA^k^** in Groups 2, 5 and 6, induced an increase in the number of sperm cells positive for **ET^x^**. Groups 5 and 6 saw lesser apoptotic effects of **CTL^q^**
Yakubu *&* Jimoh (2015)	Rats Group 1 n=5 0.5 ml **AD^m^** Group 2 Paroxetine n=5 (10mg/kg) 0.5 ml **AD^m^** Group 3 Paroxetine n=5 (10mg/kg) 0.5 ml **AD^m^** and 47 mg/kg extract Group 4 Paroxetine n=5 (10mg/kg) 0.5 ml **AD^m^** and 94 mg/kg extract Group 5 Paroxetine n=5 (10mg/kg) 0.5 ml **AD^m^** and 141 mg/kg extract	1 daily dose group 1, per 7 days 1 daily dose of antidepressant, ADm and extract, for 21 days	**Biochemical parameters:** groups 2, 3, 4 and 5 had reduced levels of total protein, sialic acid, glycogen, total cholesterol and testosterone (*p*<0.05). **Testicular analysis:** groups 3, 4 and 5 had reduced alkaline phosphatase and acid phosphatase activity, **LD^y^** and **GGT^z^**. Decreased effects with the extract, compared to Group1. Group 5 had attenuated levels of androgenic parameters when compared to Group 1.

## DISCUSSION

The effects of selective serotonin reuptake inhibitors (SSRIs) on male fertility have recently become the subject of human, animal, and *in vitro* studies. Nørr *et al*. (2016) reviewed studies published until 2014 and found that the impact of SSRIs on fertility varied depending on the specific drug and duration of use. These medications appeared to affect male fertility by significantly reducing sperm count and motility, increasing abnormal sperm morphology, and increasing DNA fragmentation. However, most human studies showed some degree of contradiction due to the primary occurrence of depression. Depression and anxiety can cause reduced libido, erectile dysfunction, and ejaculatory problems (Nørr *et al*., 2016).

This review presents a perspective based on a systematic analysis of articles published in the last decade. Database searches revealed that fluoxetine is the most studied drug regarding the effects of SSRIs on male fertility, with the majority of studies being experimental and utilizing mice or rats (Table 1).

Research on fluoxetine treatment in adult rats indicated that administering the drug at a dosage of 20 mg/kg body weight for 11 days resulted in increased individual area of Leydig cells and plasma testosterone and decreases in testicular and epididymal weight, seminal vesicle weight, seminiferous tubule length, total Leydig cell volume, daily sperm production, 17βHSD6 activity, and serum testosterone levels (Câmara *et al*., 2019). Similar outcomes were reported by Bezerra *et al*. (2019), who noted increased spontaneous contractions of the rat distal tail, potentiation of phenylephrine-induced contractions, decreased serum testosterone levels, decreased daily sperm production, lower epididymal sperm reserves, and longer epididymal transit time when rats received a daily dose of fluoxetine at 20 mg/kg. Conversely, when rats were given a daily dose of fluoxetine of 10 mg/kg for two months, testosterone levels were not lower than those observed in the control group (Erdemir *et al*., 2014). These studies conclude that the 20 mg/kg body weight dose induced the most significant changes in the male reproductive system.

In an *in vitro* study using fluoxetine at concentrations of 1, 3, and 10 µM, decreases were observed in induced contractions of the distal epididymal tail by KCl, phenylephrine, or carbachol, particularly at concentrations of 3 and 10 µM. In comparison, at 1 µM, phenylephrine-induced contractions were potentiated (Bezerra *et al*., 2019).

Effects on male offspring were noted when fluoxetine was administered to pregnant and lactating female rats, particularly in the offspring of females treated with 20 mg/kg body weight, resulting in reduced testicular (16%), epididymal (28%), seminal vesicle (18%), seminiferous tubule (17%) weight, total Leydig cell volume (30%), seminiferous tubule length (17%), daily sperm production (18%), increased individual area of Leydig cells (7%), and plasma testosterone (49%) (Monteiro Filho *et al*., 2014). Other doses led to less significant alterations in offspring, reinforcing the conclusion that the 20 mg/kg body weight dose caused the most changes in the male reproductive system.

The effect of sertraline (20 mg/kg) was studied in rats receiving daily doses for 21 days, showing increased spontaneous contractions of the distal epididymal tail, potentiation of phenylephrine-induced contractions, decreased serum testosterone levels, and decreased daily sperm production and sperm reserves in the epididymis (Bezerra *et al*., 2019). A study in men receiving daily doses of 25 or 50 mg/day for three months and a control group treated with behavioral therapy showed a significant reduction in sperm concentration (105/mL) and normal morphology percentage in the sertraline-treated group, with no significant changes in semen parameters observed in the behavioral therapy group. Sperm DNA fragmentation in the sertraline-treated group was significantly higher than in the behavioral therapy group (Akasheh *et al*., 2014). In experiments where rats received a daily dose of sertraline at 10 mg/kg for two months, there were no differences in luteinizing hormone levels, malondialdehyde, or Johnsen scores. Follicle-stimulating hormone levels were higher than in the control group, and testosterone levels were lower (Erdemir *et al*., 2014).

In experiments where rats received a daily dose of escitalopram 10 mg/kg for two months, there were no differences in luteinizing hormone levels, malondialdehyde, Johnsen scores, or follicle-stimulating hormone levels, while testosterone levels were lower (Erdemir *et al*., 2014). Rats treated with citalopram (5, 10, and 20 mg/kg) daily for 28 days showed reduced sperm concentration and normal morphology, increased sperm DNA fragmentation, testicular changes, and increased luteinizing hormone levels. Testosterone levels decreased in groups receiving 5 or 10 mg/kg, and regarding glutathione, an antioxidant marker, reduction indicated increased oxidative stress in groups receiving 10 or 20 mg/kg (Ilgin *et al*., 2017). These studies also show that the 20 mg/kg body weight dose induced the most changes in the male reproductive system.

A study on the effect of citalopram (10 mg/kg) was conducted with mice given daily doses for 35 days alone or in combination with melatonin (10 and 20 mg/kg) (Moradi *et al*., 2023). Citalopram caused gonadotoxic effects such as decreased testosterone levels and testicular histopathology alterations. The mice given citalopram with melatonin saw an improvement in spermatogenesis, with enhanced sperm count, motility, viability, morphology, and chromatin integrity. According to the authors, melatonin offers a protective effect by modulating nitro-oxidative stress and apoptosis, minimizing side effects on fertility (Moradi *et al*., 2023).

In another experiment, rats were randomized into receiving a daily dose of fluvoxamine 9 mg/kg (low therapeutic dose) or 27 mg/kg (high therapeutic dose) for 8 weeks, followed by analysis, or receiving the same daily dose for 8 weeks and being left untreated for 8 weeks, followed by analysis (Galal *et al*., 2016). The animals receiving 27 mg/kg developed leukocytosis, lymphocytosis, and monocytosis. All treated animals had hepatic, renal, and cardiac dysfunction and significant steroidogenesis-related hormone levels and spermogram alterations. Increased oxidative stress and testicular tissue apoptosis were also observed. According to the authors, these changes were reversed during the recovery period of the group kept untreated for 8 weeks before analysis. Still, the observed adverse effects must be considered when administering this drug.

A study in which rats received a daily dose of paroxetine 20 mg/kg for 2 months found no differences in luteinizing hormone, malondialdehyde, or follicle-stimulating hormone levels, while testosterone levels were lower, and the Johnsen score was significantly lower (Erdemir *et al*., 2014). The effect of paroxetine was also studied in rats receiving 10 mg/kg doses of the medication versus rats receiving the same medication at the same dose with an aqueous extract of *Carpolobia lutea* root (Yakubu & Jimoh, 2015). The authors observed that all animals receiving paroxetine showed reductions in total protein, sialic acid, glycogen, total cholesterol, and testosterone levels. Regarding testicular analysis, treated animals also showed reductions in alkaline phosphatase, acid phosphatase, lactate dehydrogenase, and gamma-glutamyl transferase activities. Conversely, groups receiving 141 mg/kg of *Carpolobia lutea* extract showed improvements in androgenic parameter levels.

A comparative study including 20 males with idiopathic infertility (6 treated with antidepressants, 4 with benzodiazepines, and 10 with antipsychotics) treated for more than three months and ten fertile untreated couples measured the levels of psychotropic drugs in semen (Mazzilli *et al*., 2021). The study participants underwent clinical and andrological examinations, including semen parameters and drug metabolite levels. Alprazolam, olanzapine, and levetiracetam showed similar semen and serum concentrations, while fluoxetine, quetiapine, and aripiprazole were detectable but had significantly lower semen levels than the serum therapeutic range. Progressive sperm motility was significantly decreased in individuals treated with psychotropic drugs compared to controls. Sperm concentration and progressive motility were reduced considerably in individuals treated with antipsychotics compared to untreated controls and individuals administered other classes of psychotropics. This indicated the possibility that antipsychotics may have more intense harmful effects than other psychotropic drugs on male fertility. Overall, the authors suggested a potential correlation between psychotropics and alterations in sperm concentration and motility (Mazzilli *et al*., 2021).

According to Beeder & Samplaski (2020), most of the negative effects associated with SSRIs appear to be reversible upon treatment cessation, although they are indicated for all cases of depression. Antioxidants assist in combating oxidative stress, and vitamins and supplements might minimize effects on semen parameters, although further studies are required (Parece, 2022).

## CONCLUSION

Considering the long-term nature of antidepressant therapy, its effects on fertility must be carefully considered. Research indicates that these drugs increase rates of DNA fragmentation and oxidative stress in the reproductive organs. They can also elevate serum LH, FSH, prolactin, and steroidogenesis hormone levels and inhibit LH binding to Leydig cells, leading to reversible suppression of spermatogenesis. They may also decrease the production of viable sperm, sperm concentration, motility, and the presence of sperm with normal morphology and cause decreases in testicular, epididymal, and seminal vesicle weight. However, despite studies pointing to the adverse effects of SSRIs, further randomized clinical and experimental research is needed to evaluate the mechanisms of spermatogenic failure and investigate the underlying causes for these effects on male fertility. Fluoxetine and sertraline have been the subject of particular attention. Other articles have evaluated the effects of substances that might mitigate the impact of continuous SSRI use on male fertility, enabling the treatment of depression without affecting one’s reproductive capacity. Nonetheless, more clinical studies on this topic are required.
